# Men and women’s hearts don’t beat the same: Epicardial mapping of Bachmann’s bundle

**DOI:** 10.1007/s12471-025-02001-x

**Published:** 2025-11-11

**Authors:** Anouk I. Freriks, Nicole L. M. de Kruijf, Mathijs S. van Schie, Vehpi Yildirim, Paul Knops, Lara M. Vos, Maryam Kavousi, Yannick J. H. J. Taverne, Natasja M. S. de Groot

**Affiliations:** 1 Department of Cardiology, Erasmus Medical Center, Rotterdam, The Netherlands; 2 Department of Epidemiology, Erasmus Medical Center, Rotterdam, The Netherlands; 3 Department of Cardiothoracic Surgery, Erasmus Medical Center, Rotterdam, The Netherlands; 4 Department of Microelectronics, Signal Processing Systems, Faculty of Electrical Engineering, Mathematics and Computer Sciences, Delft University of Technology, Delft, The Netherlands; 5 Unit Translational Electrophysiology, Department of Cardiology, Erasmus Medical Center, Rotterdam, The Netherlands

**Keywords:** Atrial fibrillation, Bachmann’s bundle, Electrophysiological properties, Sex difference

## Abstract

**Background:**

There is increasing evidence that presentation, progression, and management of atrial arrhythmias, such as atrial fibrillation (AF), differ between women and men. Bachmann’s bundle (BB) is the main route for interatrial conduction, and sex-related differences in structural and electrical remodeling of BB may contribute to differences in AF development between women and men.

**Objective:**

Investigate whether sex differences in the electrophysiological properties of BB assessed by high-resolution and density maps exist in patients with AF.

**Methods:**

Sinus rhythm at BB was recorded for 5 s during cardiac surgery. Potential voltage, low-voltage area (LVA), conduction heterogeneity, unipolar potential morphology, and conduction velocity were assessed for both men and women.

**Results:**

The study population consisted of 108 patients (73 men, 35 women). Women had significantly lower potential voltages (5th percentile: 0.7 mV [0.6–1.0] vs 1.1 mV [0.6–1.4], *p* = 0.028), more LVAs (10.8% [4.6–19.7] vs 4.3% [2.2–11.7], *p* = 0.012) and more long double potentials (11.1% [3.6–13.5] vs 5.0% [1.0–10.3], *p* = 0.015) compared to men.

**Conclusions:**

We observed sex-related differences in the electrical remodeling of BB in AF patients. Women have a higher proportion of low voltage potentials, and more abnormal potential morphologies compared to men. These findings may reflect sex-specific differences in the underlying substrate of AF at BB.

**Supplementary Information:**

The online version of this article (10.1007/s12471-025-02001-x) contains supplementary material, which is available to authorized users.

## What’s new?

- This is the first study to investigate sex differences in the electrophysiological properties of Bachmann’s bundle in patients with a history of atrial fibrillation.
- Post-menopausal women have significantly lower potential voltages, more low-voltage areas, and more long double potentials compared to men.
- These findings may indicate sex-specific differences in the underlying substrate of atrial fibrillation at Bachmann’s bundle.

## Introduction

There is increasing evidence that presentation, progression, and management of atrial arrhythmias, such as atrial fibrillation (AF), differ between women and men [1]. Although AF occurs more frequently in men, women with AF tend to experience more severe symptoms, a worse quality of life, and a higher risk of complications such as stroke and heart failure [2]. These observations suggest that the pathophysiology of AF may differ between men and women.

Prior studies demonstrated that sex-based differences are already present during sinus rhythm (SR) [3, 4]. A prolonged P‑wave duration can be a sign of an interatrial conduction block. It is assumed that this is caused by a complete line of block at Bachmann’s bundle (BB) [5]. BB is the main route for interatrial conduction, which runs from the superior cavo-atrial junction of the right atrium to the left atrial appendage. It is thought that the parallel organization of the muscle fibers at BB facilitates longitudinal conduction, enabling wavefronts to spread faster in the longitudinal direction [6]. Because of this parallel structure, even small structural changes can disrupt fiber organization. This structural remodeling can increase non-uniformity of conduction anisotropy and local directional heterogeneity at BB, making it more susceptible to conduction disorders. Notably, these conduction disorders are already present during sinus rhythm (SR), highlighting the early vulnerability of BB to remodeling [7]. Several studies showed that BB is indeed a predilection site for conduction disorders, which are associated with an increased risk of developing AF [8–11]. However, it is unknown whether in patients with AF, there are sex differences in remodeling of BB.

The aim of this study is therefore to determine whether sex differences in the electrophysiological properties of BB assessed by high-resolution and density maps exist in patients with AF.

## Methods

### Study population

The study population consisted of patients aged 18 years and older with AF undergoing elective open heart surgery for aortic, coronary artery, and/or valvular heart disease. All AF patients who were in SR at the moment of the mapping procedure were selected from the existing databases of the Halt&Reverse and QUASAR studies (MEC2010-054&MEC2014-393) [12, 13]. Written informed consent was obtained from all patients prior to the surgery, and clinical information was retrieved from their electronic medical records.

### Mapping procedure

**Fig. 1:**
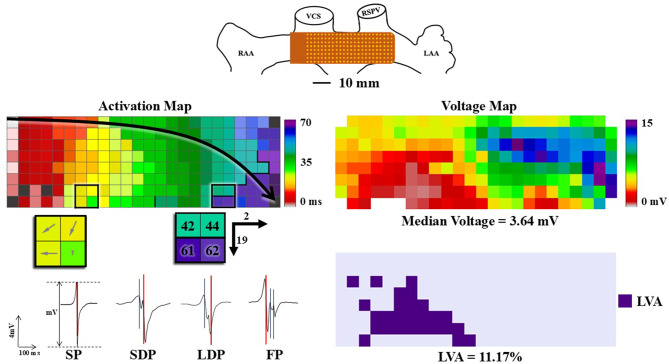
High-resolution epicardial mapping of BB. Upper panel: Schematic overview of the BB mapping position. Upper left panel: Example of a colour-coded activation map. The arrow shows the main conduction direction. The box on the left shows a CV map, with arrows showing the direction of activation, a larger arrow indicates a faster CV. The box on the right shows an example of CB. Lower left panel: Example of the different potential morphology types. In the left signal an example of the peak-to-peak voltage is shown. Upper right panel: Example of a median voltage map. Lower right panel: Example of a low voltage map. (*BB* Bachmanns Bundle, *CB* Conduction block, *CV* Conduction velocity, *FP* Fractionated potential, *LAA* Left atrial appendage, *LAT* Local activation time, *LDP* Long double potential, *LVA* Low voltage area, *RAA* Right atrial appendage, *RSPV* Right superior pulmonary vein, *SDP* Short double potential, *SP* Single potential, *TAT* Total activation time, *VCS* Vena cava superior)

Epicardial high-resolution mapping was performed prior to extracorporeal circulation as described previously [14]. A reference electrode was temporarily attached to the right atrial free wall. The mapping approach consists of placing a 128- or 192-electrode array (interelectrode distances of 2 mm; electrode diameter, respectively 0.45 and 0.65 mm) at BB with the distal side of the electrode array on the border of the left atrial appendage and the proximal side of the array towards the superior caval vein (Fig. 1, upper panel).

### Data analysis

Using custom-made software, local activation times, defined as the moment of the steepest negative deflection of the unipolar potentials, were automatically annotated to construct color-coded activation maps, as demonstrated in the upper left panel of Fig. 1. Interelectrode conduction times (CTs) were used to construct the relative frequency distribution histograms. Conduction delay (CD) was defined as CT differences of 7–11 ms and conduction block (CB) as CT ≥ 12 ms [15, 16]. The amount, number, and maximum length of lines of CB and continuous (c)CDCB were analyzed. Signal morphology according to the number of deflections, including single- (one deflection, SP), short double- (two deflections with a distance < 15 ms, SDP), long double- (two deflections with a distance ≥ 15 ms, LDP) or fractionated potentials (≥ 3 deflections, FP) [17]. Typical examples of each of these potentials are shown in the left lower panel of Fig. 1. For each type of unipolar potential, we defined the prevalence as a percentage of all unipolar potentials measured. For each patient, the median, 5th percentile (P5), and 95th percentile (P95), and the potential voltage variance defined as the difference between the P5 and P95 of all unipolar potential voltages, were calculated. Unipolar potential voltage was defined as the peak-to-peak voltage of the steepest negative deflection. Low potential voltage areas (LVAs) were defined as areas with a peak-to-peak amplitude < 1 mV. An example of a unipolar and low-potential voltage map is shown in the right panel of Fig. 1.

Using the technique as previously described by Van Schie et al. [18], the conduction velocity (CV) was computed as an average of estimated velocity vectors between adjacent electrodes in the longitudinal, transverse, and diagonal direction to calculate the median, P5 and the P95 of all CVs; the variance in CV was calculated by subtracting the P5 from the P95 CV. Local directional heterogeneity (LDH) was defined as the proportion of CV vectors that were indicated as heterogeneous, based on the method by Van Schie et al. [6]. A local CV vector was classified as directional heterogeneous when the local propagation angle differed more than 50% from the mean of all surrounding local propagation angles and/or the local speed was at least 50% slower than the geometric mean of all surrounding velocities. Heterogeneity (LDH) was then calculated as the proportion of all CV vectors.

All electrophysiological parameters were calculated per patient, and group comparisons between women and men were performed using these patient-level values. An overview of all parameters used for analysis can be found in Electronic Supplementary Material Table S1.

### Statistical analysis

All data were tested for normality. Continuous data were presented as mean (standard deviation [SD]), unless they were not normally distributed, then they were presented as a median (interquartile range [IQR]). If the data were normally distributed, a *t*-test was used, and a Mann-Whitney U test was used for skewed data. A *p*-value < 0.05 was considered statistically significant. Categorical data were presented as an absolute number (percentage) and analyzed with a chi-squared test.

## Results

### Study population

A total of 108 patients were included; baseline characteristics of the men (*N* = 73, age 70.3 years [65.3–76.9]) and women (*N* = 35, age 71.3 years [67.7–76.5]) are summarized separately in Tab. 1 and did not differ between the two groups. Also, the median AF duration was comparable between the sexes (men 0.7 years [0.3–3.8] vs women 0.6 years [0.3–6.2], *p* > 0.05), and the distribution of AF types (paroxysmal, persistent, and long-standing persistent) was also similar between men and women (all *p* > 0.05).

**Table 1:** Baseline characteristics

	Men (*n* = 73)	Women (*n* = 35)	*P*-value
*Age*	70.3 (65.3–76.9)	71.3 (67.7–76.5)	0.646
*BMI*	26.3 (24.3–29.1)	27.3 (23.2–31.9)	0.247
*AF duration (y)*	0.7 (0.3–3.8)	0.6 (0.3–6.2)	0.377
*Hypertension (%)*	40 (54.8)	21 (60.0)	0.762
*Dyslipidemia (%)*	20 (27.4)	11 (31.4)	0.837
*Diabetes Mellitus (%)*	11 (15.1)	9 (25.7)	0.285
*LA Dilatation (%)*	39 (53.4)	17 (48.6)	0.807
*Underlying heart disease (%)*			
– IHD	21 (28.8)	8 (22.9)	0.677
– VHD	29 (39.7)	21 (60.0)	0.077
– IVHD	17 (23.3)	3 (8.6)	0.115
– Aorta	0 (0.0)	1 (2.9)	0.706
– Other	6 (8.2)	2 (5.7)	0.942
*Left ventricular function (%)*			
– Normal	57 (78.1)	27 (77.1)	1.000
– Moderate	7 (9.6)	1 (2.9)	0.391
– Mild	9 (12.3)	7 (20.0)	0.447
*AF Type (%)*			
– Paroxysmal	52 (71.2)	27 (77.1)	0.677
– Persistent	18 (24.7)	8 (22.9)	1.000
– Longstanding Persistent	3 (4.11)	0 (0.0)	0.555

*AF* Atrial fibrillation, *BMI* Body mass index, *IHD* Ischemic heart disease, *IVHD* Ischemic and valvular heart disease, *VHD* Valvular heart disease, *Y* Years

### Mapping data characteristics

During 5 s of SR, a total of 120,420 unipolar potentials were recorded, with 81,495 potentials recorded in men and 38,925 in women. The total activation time over BB was not significantly different between men and women (56 ms [42–74] vs 62 ms [50.5–74.5], *p* > 0.05). Similarly, the median cycle length was comparable between the sexes (men 875 ms [768.5–1060.5] vs women 862.5 ms [744.3–993.8], *p* > 0.05).

### Sex differences in conduction heterogeneity

Fig. S1 illustrates the median CV values per patient for men and women separately. In men, local CV ranged from 49.8 to 126.9 cm/s and in women from 56.8 to 103.2 cm/s. A trend towards lower CV was observed in women (79.8 cm/s [74.2–89.2]) compared to men (89.9 cm/s [76.3–94.2]), but this difference did not reach statistical significance (*p* = 0.053). The P5 and P95 of the CV histogram, variation in CV, and LDH were not significantly different between the sexes (all *p* > 0.05) (see Electronic Supplementary Material Tab. S2).

The frequencies of CB and cCDCB were similar between men and women (both *p* > 0.05). Likewise, the maximum lengths of CB and CDCB lines were comparable across sexes (both *p* > 0.05) (Tab. S3).

### Sex differences in unipolar potential morphology

Women had a significantly higher number of LDPs compared to men (11.1% [3.6–13.5] vs 5.0% [1.0–10.3], *p* = 0.015). Women had a lower percentage of single potentials compared to men, although this difference was not significant (71.5% [63.7–79.5] vs 77.0% [62.8–85.6], *p* = 0.325). The number of SDPs, FPs, and the duration of all double and complex potentials were comparable for men and women (all *p* > 0.05). An overview of the occurrence of the different types of potential morphology in both sexes can be found in Tab. 2.

**Table 2:** Unipolar potential morphologies

	Men	Women	*P*-value
Singles (%)	77.0 (62.8–85.6)	71.5 (63.7–79.5)	0.325
SDP (%)	14.0 (8.1–21.1)	12.2 (7.3–17.8)	0.419
LDP (%)	5.0 (1.0–10.3)	11.1 (3.6–13.5)	0.015*
Fractionation (%)	3.1 (0.8–7.0)	2.9 (1.8–5.7)	0.730
LDP + Fractionated potentials (%)	9.0 (2.0–17.3)	14.6 (6.9–18.8)	0.061
Duration SDP (ms)	8.0 (7.0–9.0)	8.0 (7.0–9.5)	0.611
Duration LDP (ms)	19.0 (17.0–21.5)	20.0 (18.0–23.0)	0.144
Duration fractionated potentials (ms)	18.0 (14.0–22.5)	20.5 (15.0–25.0)	0.233

*LDP* Long double potential, *SDP* Short double potential

### Sex differences in unipolar potential voltages

**Fig. 2:**
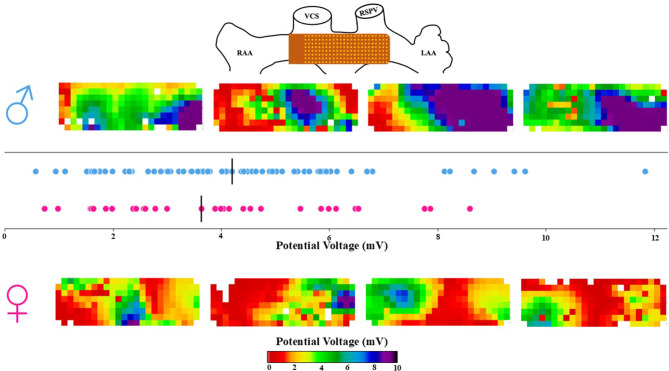
The upper panel illustrates the position of the electrode array at BB during the epicardial mapping procedure. Below, four examples of typical voltage maps from men are presented, with corresponding examples from women displayed at the bottom. The central panel shows a comparison of unipolar median voltages for all men and women, with the median values indicated by black lines. (*BB* Bachmanns Bundle)

Figure 2 presents typical unipolar potential voltage maps for men (upper panel) and women (lower panel). These maps demonstrate that there is large variability in potential voltages across BB in both sexes and that unipolar potential voltages are higher in men compared to women. The graph in the center illustrates the distribution of median unipolar potential voltages for each individual man and woman. As shown, the median potential voltage for men ranged from 0.6 to 11.8 mV, while for women it ranged from 0.7 to 8.6 mV. Median potential voltages in women were lower, but this difference was not statistically significant (*p* > 0.05).

However, the P5 of all potential voltages was significantly lower in women (0.7 mV [0.6–1.0]) compared to men (1.1 mV [0.6–1.4], *p* = 0.028), whereas the P95 and voltage variance were comparable between the two groups (both *p* > 0.05) (Tab. 3).

**Table 3:** Unipolar voltages

	Men	Women	*P*-value
Median Voltage (mV)	4.2 (2.9–5.5)	3.6 (2.0–5.1)	0.203
LVA (%)	4.3 (2.2–11.7)	10.8 (4.6–19.7)	0.012*
Voltage P5 (mV)	1.1 (0.6–1.4)	0.7 (0.6–1.0)	0.028*
Voltage P95 (mV)	9.6 (6.5–12.5)	9.1 (6.6–11.8)	0.689
Voltage Range (mV)	8.2 (5.9–11.1)	8.5 (6.0–11.0)	0.937

*LVA* Low Voltage Area, *P5* 5th percentile, *P95* 95th percentile

### Sex differences in low voltage areas

**Fig. 3:**
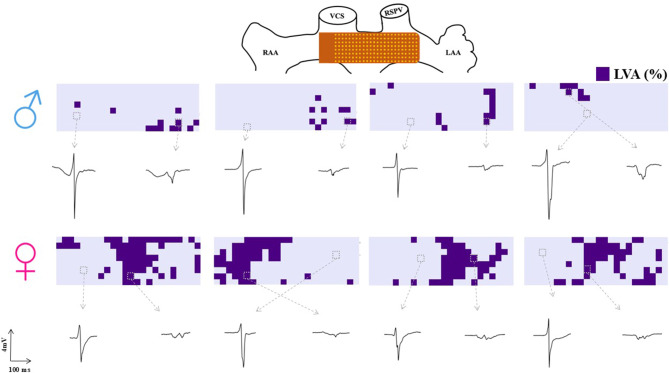
The upper panel illustrates the position of the electrode array at BB during the epicardial mapping procedure. Below, four examples of typical low-voltage maps from men are shown, each accompanied by corresponding signals: a normal voltage signal (left) and a low-voltage signal (right). At the bottom, four low-voltage maps from women are displayed, also with corresponding normal and low-voltage signals. (*BB* Bachmanns Bundle)

Low potential voltage maps of 4 typical men and women are shown in Fig. 3, illustrating differences in the extent of LVAs. Below the potential voltage maps, examples of potentials with normal and low voltages are demonstrated. In these example maps, men exhibit fewer LVAs, which are also more dispersed across BB. Women show a more concentrated distribution of LVAs, primarily located in the middle and right of BB. In line with this observation, we found in the entire study population that women have a significantly higher percentage of LVAs compared to men (10.8% [4.6–19.7] vs 4.3% [2.2–11.7], *p* = 0.012).

## Discussion

### Key findings

With high-resolution epicardial mapping, we revealed significant sex-based differences in the electrophysiological properties of BB in patients with AF. We found that women have notably lower potential voltages and more LVAs compared to men. Additionally, abnormal potential morphologies were more frequently recorded in women than in men.

### Unipolar potential voltages at BB in patients with and without AF

Although the P‑wave duration on the electrocardiogram is significantly longer in men, we found that BB is more affected in women. This contrast confirms the findings by Mouws et al., who showed that the P‑wave duration does not reflect the interatrial conduction at BB [19]. Several studies discovered differences in unipolar potentials in patients with AF at BB, suggesting an important role for BB in the development and perpetuation of AF. For example, in patients with mitral valve disease, LVAs were more prevalent in patients with a history of AF [11]. Moreover, patients with AF exhibit lower unipolar potential voltages compared to patients without a history of AF [20]. Teuwen et al. [6] found no difference in mean unipolar voltage between patients with and without AF in the presence of coronary artery disease. However, they did find an association between women and lower averaged potential voltages. In our study, the percentage of LVAs observed at BB was more than twice as high in women compared to men. This sex-based difference aligns with the observation by Teuwen et al. who found lower potential voltages in women but contrasts with the findings of Veen et al. who found no difference in LVAs between men and women without AF [3], suggesting that this difference arises during the development of AF. This could be explained by AF-induced remodeling rather than pre-existing substrate differences, according to the principle that “AF begets AF”, recurrent or sustained AF promotes structural and electrical remodeling [21].

### Atrial remodeling and LVAs at BB

The larger prevalence of LVAs in women could be linked to underlying differences in atrial remodeling. From an earlier study in patients with longstanding persistent AF, it is known that women have a higher degree of fibrosis compared to men, which can be explained by an upregulation of the transforming growth factor β/Smad3 signaling pathway in women [22]. This higher degree of fibrosis may help explain the significantly larger prevalence of LVAs observed in women, as LVAs often serve as markers of fibrotic tissue [23]. Another theory suggests that women have higher levels of several adipokines, cytokines secreted by the adipose tissue that promote inflammation and fibrotic remodeling [24]. It is unlikely that fibrotic remodeling is limited to BB. However, we hypothesize that the effect may be more extensive in BB due to its unique parallel fiber organization, which may make this structure more susceptible to remodeling compared to other atrial regions [25]. This structural susceptibility could make BB more prone to fibrotic remodeling compared to other atrial regions, potentially linking LVAs to its increased fragility [26]. A comparison with other atrial regions is needed to confirm this hypothesis in future studies.

Over the past years, LVAs have gained increased attention as a target for AF ablation procedures [27]. Despite the recognized role of LVAs in arrhythmogenesis, current ablation procedures do not specifically target BB. Given the higher incidence of LVAs at BB in women, BB may represent a promising, though currently underexplored, ablation target in this population [28]. Further research is necessary to investigate whether targeted ablation at or around BB could offer a benefit in selected patients.

### Unipolar potential morphology at BB in patients with and without AF

Veen et al. found that women without AF have more SDPs, while the amount of LDPs remained similar between the sexes [4]. In contrast, we observed a comparable number of SDPs between the sexes, but women had significantly more LDPs. SDPs may reflect normal physiological heterogeneity in the atrial wall, whereas LDPs indicate regions of asynchronous tissue activation—either in the 2D or 3D plane—, suggesting disrupted or delayed conduction [29]. Since we found no difference in CB in the epicardial plane between the sexes, the observed increase in LDPs in women may be attributed to asynchronous activation caused by CB lines of atrial layers deeper within the atrial wall [30].

### Effect of hormones on AF development

The population in our study was relatively old, and most women were likely postmenopausal. The menopause marks a decline in estrogen levels, reducing its potential protective effect against AF in women by modulating potassium and calcium channels [31, 32]. The decrease in estrogen may also cause an increase in LVAs by promoting atrial fibrosis due to elevated postmenopausal follicle-stimulating hormone levels [33]. This is in line with our findings that women had twice as much LVAs compared to men.

### Limitations

This study only included patients undergoing cardiac surgery. Since this is more prevalent in men, our study has a higher proportion of men, potentially underestimating sex-specific differences. Future research with a matched study population is crucial to better understand sex-specific differences in electrophysiology. The relatively small sample size in this study may limit the statistical power to detect subtle differences or perform robust multivariable analyses. Moreover, our findings are particularly relevant to post-menopausal women and may not necessarily apply to younger populations or individuals in different age groups. We did not take the atrial size into account, since pre-operative echocardiograms were not available for all patients.

## Conclusion

We observed sex-related differences in the electrical remodeling of BB in patients with AF. Women have a higher proportion of low voltage potentials, and more abnormal potential morphologies compared to men. These findings may reflect sex-specific differences in the underlying substrate of AF at BB. Nevertheless, uncontrolled factors may have contributed to the observed differences, underscoring the need for confirmation in larger cohorts. Electrophysiological data from women remain scarce, and our findings highlight the importance of including more women in electrophysiological studies to demonstrate sex differences in arrhythmogenic substrates.

## Supplementary Information

Table S1 Overview of all parameters included in the analysis

Table S2 Conduction heterogeneity

Table S3 Conduction disorders total electrode

**Supplemental Fig. 1 **Median CV (cm/s) per patient over BB compared between men and women, ranked from lowest to highest CV

## Data Availability

The data underlying this article will be shared on reasonable request to the corresponding author.
