# Supplementary material for: Men and women’s hearts don’t beat the same: Epicardial mapping of Bachmann’s bundle
Source: Neth Heart J. 2025 Nov 11;33(12):421–8. doi: 10.1007/s12471-025-02001-x (PMC12638521; doi:10.1007/s12471-025-02001-x)
Supplement: Supplementary file 1 — Table S1 Overview of all parameters included in the analysis [file 12471_2025_2001_MOESM1_ESM.docx]

**Supplemental Table 1** Overview Parameters

| Parameter | Abbreviation | Definition | Use/Purpose |  |
| --- | --- | --- | --- | --- |
| Total activation time (ms) | TAT | Time between earliest and latest activation across the mapped area | Reflects conduction across the atria: increased values may indicate conduction slowing or block | 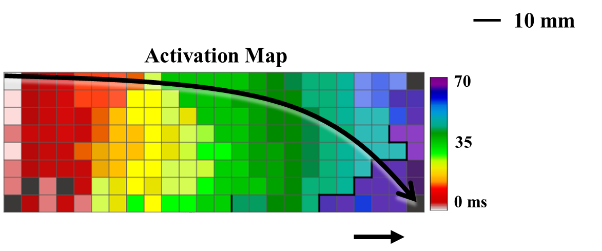TAT = 68 ms |
| Cycle Length (ms) | CL | Time between two consecutive atrial beats | Reflects atrial activation rate and can indicate arrhythmia dynamics | 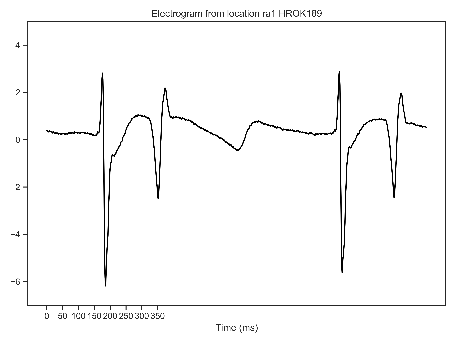 |
| Conduction Velocity (cm/s) | CV | Speed of activation wavefront propagation across the atrial tissue | Identifies regions of slow conduction, can be important in AF substrate mapping | 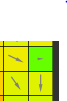 |
| Local directional heterogeneity | LDH | Proportion of CV vectors that were indicated as heterogeneous in angle or speed | Identifies local heterogeneity in conduction direction (6) | \| 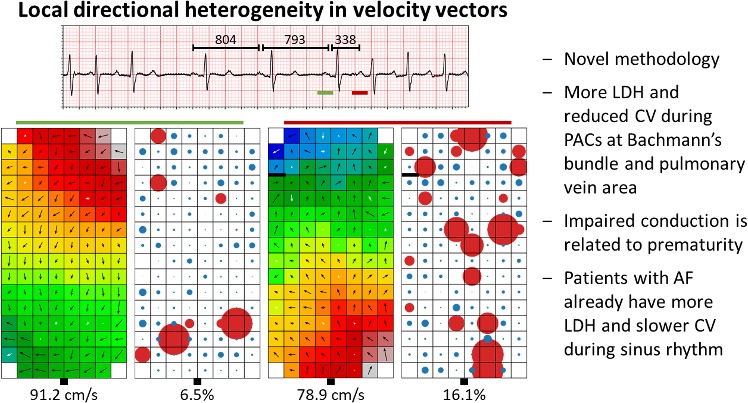p \| \| \| \| \| --- \| --- \| --- \| --- \| \| 91.2 cm/s \| 6.5% \| 78.9 cm/s \| 16.1% \| |
| Conduction block (%, mm) | CB | Activation delay >12 ms between adjacent electrodes. Can be expressed as the total area or length of the CB line. | Indicates extent of conduction disturbances; associated with arrhythmia risk (15,16) | 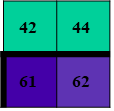 |
| Conduction Delay Conduction Block (%, mm) | CDCB | Areas of uninterrupted conduction delay and CB. Can be expressed as the total area or length of the CDCB line. | Measures potential conduction corridors or anchor points for reentry circuits. (15,16) | 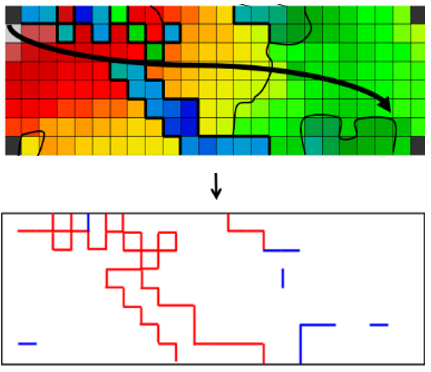  CD  CB |
| Single Potentials (%) | SP | Electrograms with a single negative deflection | Indicates normal, uniform conduction. (17) | 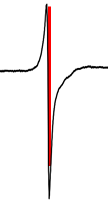 |
| Short Double Potentials | SDP | Two negative deflections with a distance of <15 ms | Normal difference in conduction between the endocardial and epicardial layers of the atrial wall. (17) | 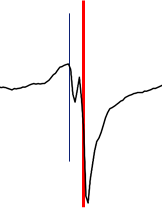 |
| Long Double Potentials | LDP | Two negative deflections with a distance of >15 ms | Reflects structural or functional conduction disorders and may indicate atrial regions important for sustaining AF. (17) | 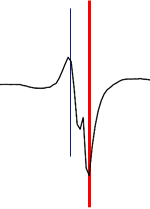 |
| Fractionated Potentials (%, duration) | FP | Three or more negative deflections. Can be expressed as percentage or duration between the first and last potential in a fractionated electrogram | Identifies areas of asynchronous conduction, often due to structural or functional heterogeneity; helpful in locating arrhythmogenic zones (17) | 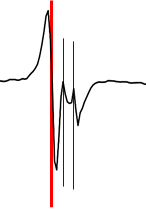 |
| Median Voltage (mV) | - | Median unipolar voltage of all potential voltages in a mapped region | Describes overall tissue excitability or integrity | 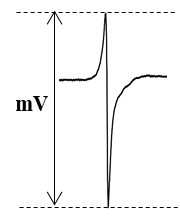 |
| Low Voltage Area (%) | LVA | Area with a voltage below <1.0 mV | Identifies scarred or fibrotic tissue; commonly used in substrate mapping (11) | 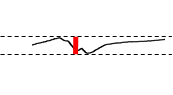  0.33 mV |
| Voltage 5^th^ percentile (mV) | Voltage P5 | 5^th^ percentile of all potential voltages | Captures the lowest voltage zones | 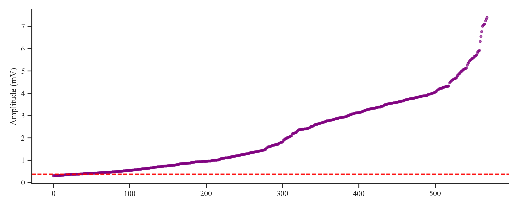 |
| Voltage 95^th^ percentile (mV) | Voltage P95 | 95^th^ percentile of all potential voltages | Identifies regional variations in voltages | 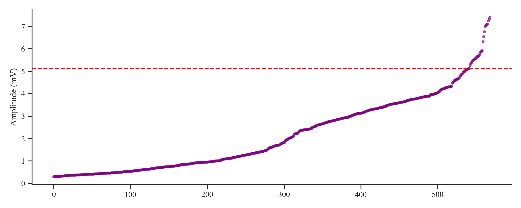 |
| Voltage Range (mV) | - | Difference between the voltage P95 and voltage P5 | Indicates tissue heterogeneity; useful for assessing electrical dispersion in voltages | 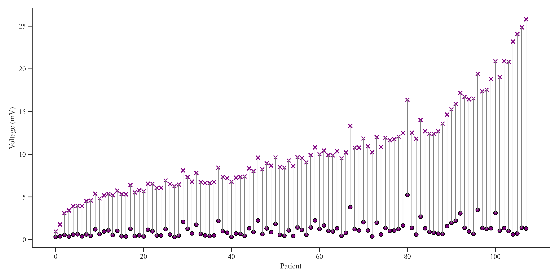 |
