# Supplementary material for: Men and women’s hearts don’t beat the same: Epicardial mapping of Bachmann’s bundle
Source: Neth Heart J. 2025 Nov 11;33(12):421–8. doi: 10.1007/s12471-025-02001-x (PMC12638521; doi:10.1007/s12471-025-02001-x)
Supplement: Supplementary file 2 — Table S2 Conduction heterogeneity [file 12471_2025_2001_MOESM2_ESM.docx]

**Supplemental Table 2** Conduction heterogeneity

|  | Men | Women | P-value |
| --- | --- | --- | --- |
| **TAT (ms)** | 56.0 (42.0 – 74.0) | 62.0 (50.5 – 74.5) | 0.224 |
| **CL (ms)** | 875.0 (768.5 – 1060.5) | 862.5 (744.3 – 993.8) | 0.545 |
| **CV (cm/s)** | 89.9 (76.3 – 94.2) | 79.8 (74.2 – 89.2) | 0.053 |
| **CV P5 (cm/s)** | 23.6 (16.4 – 29.0) | 21.4 (16.8 – 28.6) | 0.928 |
| **CV P95 (cm/s)** | 155.3 (146.6 – 162.6) | 148.3 (143.5 – 158.4) | 0.059 |
| **CV Range (cm/s)** | 131.7 (122.1 – 141.9) | 128.3 (115.5 – 138.4) | 0.117 |
| **Slow CV (cm/s)** | 7.6 (5.4 – 12.5) | 8.2 (5.3 – 10.4) | 0.844 |
| **LDH (%)** | 22.1 (18.2 – 25.7) | 20.9 (18.3 – 26.5) | 0.923 |

*CL = Cycle length; CV = Conduction velocity; LDH = Local directional heterogeneity; P5 = 5^th^ percentile; P95 = 95^th^ percentile; TAT = Total activation time*
