# Supplementary material for: Men and women’s hearts don’t beat the same: Epicardial mapping of Bachmann’s bundle
Source: Neth Heart J. 2025 Nov 11;33(12):421–8. doi: 10.1007/s12471-025-02001-x (PMC12638521; doi:10.1007/s12471-025-02001-x)
Supplement: Supplementary file 3 — Table S3 Conduction disorders total electrode [file 12471_2025_2001_MOESM3_ESM.docx]

**Supplemental Table 3** Conduction disorders total electrode

|  | Men | Women | P-value |
| --- | --- | --- | --- |
| **CB (%)** | 3.5 (1.4 – 5.7) | 4.5 (3.1 – 6.5) | 0.104 |
| **CDCB (%)** | 8.0 (4.8 – 13.2) | 10.7 (6.6 – 12.4) | 0.335 |
| **Max length CB (mm)** | 18.0 (12.7 – 29.7) | 20.0 (15.0 – 28.0) | 0.717 |
| **Max length CDCB (mm)** | 33.9 (23.3 – 48.0) | 28.0 (20.0 – 43.0) | 0.229 |

*CB = Conduction block; CDCB = Conduction delay and conduction block*
