# Supplementary figures and images for: Men and women’s hearts don’t beat the same: Epicardial mapping of Bachmann’s bundle
Source: Neth Heart J. 2025 Nov 11;33(12):421–8. doi: 10.1007/s12471-025-02001-x (PMC12638521; doi:10.1007/s12471-025-02001-x)

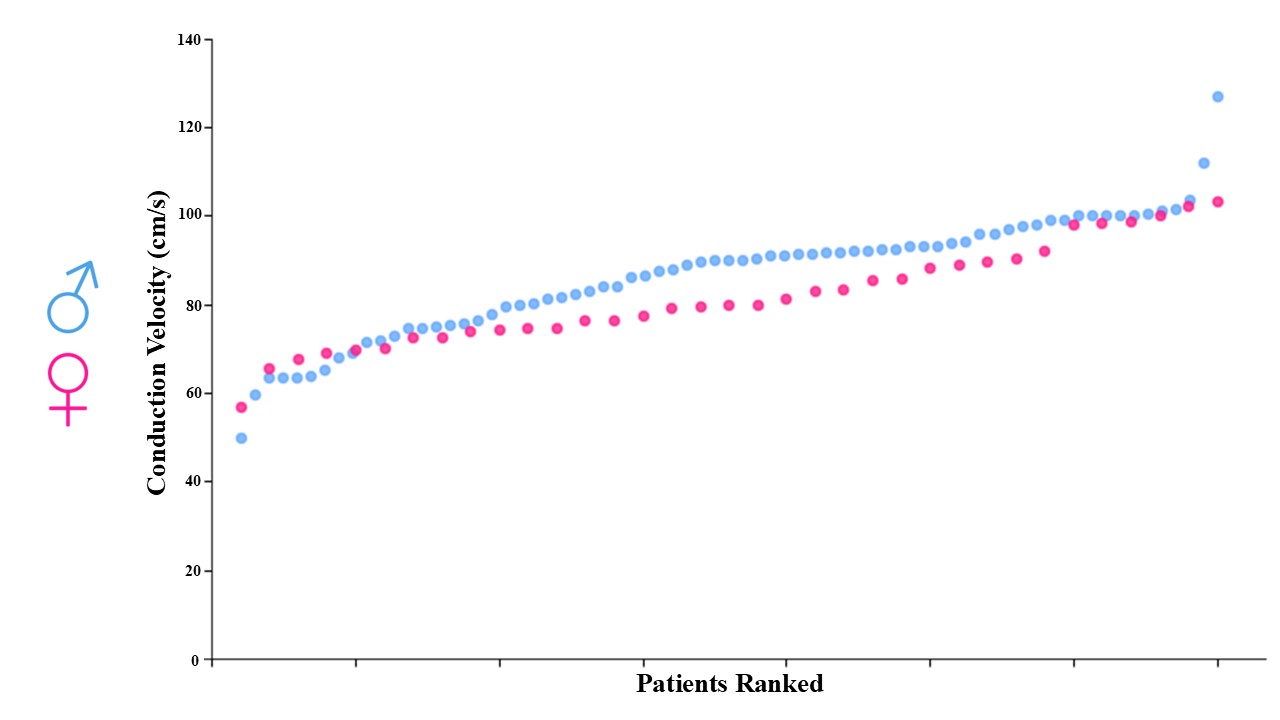

Supplement: Supplementary file 4 — Supplemental Fig. 1 Median CV (cm/s) per patient over BB compared between men and women, ranked from lowest to highest CV [file 12471_2025_2001_MOESM4_ESM.jpg]
